# Seasonal Temperature Variation Shapes Anthocyanin Composition and Berry Coloration in ‘Summer Black’ Grape

**DOI:** 10.3390/plants15162501

**Published:** 2026-08-18

**Authors:** Chengyue Li, Lifang Chen, Zihan Zhou, Runru Liu, Hongfei Lin, Congqiao Wang, Cuilan Ma, Zhipeng Qiu, Dongliang Qiu

**Affiliations:** 1 College of Horticulture, Key Laboratory Ministry of Education for Genetics, Breeding and Multiple Utilization of Crops, Fujian Agriculture and Forestry University, Fuzhou 350002, China; huajiao529@126.com (C.L.); 13509319672@163.com (L.C.); 18057272386@163.com (Z.Z.); 15753723175@163.com (R.L.); linhongfei2001@126.com (H.L.); wcq13313983179@126.com (C.W.); cuilanm@fafu.edu.cn (C.M.); 2 Lunong Agricultural Technology Co., Ltd., Xiamen 361100, China

**Keywords:** anthocyanin component, *F3′H*/*F3′5′H*, grapes, sensors, temperature

## Abstract

Berry coloration in ‘Summer Black’ grape is strongly influenced by anthocyanin composition. This study investigated how seasonal temperature variation affects anthocyanin profiles and berry coloration. Winter berries accumulated significantly higher anthocyanin levels than summer berries, with total anthocyanin content reaching 2405.38 μg/g FW (fresh weight), compared with 1440.94 μg/g FW in summer berries. Winter berries exhibited a distinct pigment profile enriched in trihydroxylated anthocyanins, and delphinidin accumulation was 2.27-fold higher than that in summer berries at the final developmental stage. Lower winter night temperatures were associated with higher *F3′5′H* expression. In contrast, higher summer night temperatures were associated with increased *F3′H* expression and cyanidin accumulation, resulting in a greater proportion of dihydroxylated anthocyanins and lighter berry coloration. These findings suggest that seasonal temperature variation, particularly night temperature under the tested double-cropping conditions, is closely associated with anthocyanin composition through differential regulation of the *F3′H* and *F3′5′H* branches of the flavonoid pathway, providing insights into the environmental regulation of berry pigmentation and quality formation in grape.

## 1. Introduction

Table grape (*Vitis vinifera* L.) is a globally important fruit crop, valued for its rich nutritional content and notable health-promoting effects. Grape berries and their derived products are rich dietary sources of anthocyanins—natural pigments with strong antioxidant activity and potential benefits in supporting human health and preventing chronic diseases [1]. In addition to their health-related functions, anthocyanins are a key quality trait in grapes, contributing significantly to berry coloration, ripening, and overall commercial value.

The biosynthesis of anthocyanins, including their compositional diversity and the underlying structural genes, has been extensively characterized in grapevines [2]. Previous studies have demonstrated that the transcriptional ratio of *F3′H* to *F3′5′H* expression significantly influences anthocyanin composition [3,4,5]. Compared with red grape varieties, blue- and purple-skinned cultivars exhibit markedly higher levels of trihydroxylated anthocyanins than dihydroxylated forms [6]. In dark-skinned grape cultivars such as ‘Summer Black’, delphinidin- and malvidin-based anthocyanins predominate, whereas red-skinned cultivars like ‘Red Globe’ show higher relative abundance of cyanidin- and petunidin-derived pigments [7,8,9].The structural genes in this pathway are broadly classified into early and late biosynthetic genes, with *CHS*, *CHI*, *F3H*, and *F3′H* mainly involved in the synthesis of flavonoids and flavonols, while *DFR*, *ANS*, and *F3′5′H* catalyze the downstream conversion into anthocyanins and proanthocyanidins [10,11,12,13].

Recent meteorological studies indicate a continuing rise in global temperatures, while night warming has been particularly pronounced [14]. Anthocyanin accumulation in grape berries is strongly influenced by environmental conditions, particularly temperature and light [15,16,17,18,19,20]. Anthocyanin biosynthesis is promoted by favorable climatic conditions, typically characterized by extended daylight duration, moderate day temperatures, and cool night temperatures (15–20 °C) [21,22]. Among these factors, low temperatures and high light intensity are particularly effective in stimulating pigment formation. Temperature not only exerts a direct influence on grapevine growth but also acts synergistically with light to regulate various metabolic processes, including the biosynthesis of secondary metabolites [23,24]. The experimental results of Yamane et al. indicated that the optimal temperature for anthocyanin accumulation in Aki Queen (*Vitis labrusca* × *V. vinifera*) was approximately 20 °C, whereas anthocyanin concentration declined sharply at 30 °C [25]. Both ‘Malbec’ and ‘Bonarda’ grape berries exhibited a significant reduction in total anthocyanin content when the average temperature increased from approximately 22–23 °C to 24–26 °C [26]. In ‘Pinot Noir’ grapes (*Vitis vinifera*), berries exposed to high temperatures of 35 °C exhibit lower anthocyanin concentrations compared with those maintained at 25 °C [27]. Under 35/25 °C (day/night) conditions, ‘Malbec’ and ‘Pinot Noir’ berries showed markedly reduced total anthocyanin concentrations at veraison (the onset of berry ripening) and harvest compared with 25/15 °C, primarily due to decreased delphinidin-, cyanidin-, petunidin-, peonidin-, and malvidin-based derivatives [28]. Understanding how these factors influence anthocyanin production is crucial for enhancing fruit quality. Such effects are particularly evident in double-cropping systems, where grapes are cultivated in two distinct seasons—summer (February–June) and winter (August–December)—allowing for two harvests per year under markedly different climatic conditions [29,30,31]. Notably, grapes harvested in the winter season often exhibit superior quality attributes and higher anthocyanin concentrations [32,33,34]. Advancements in IoT-based monitoring and control enable agricultural greenhouses to execute real-time modifications and continuous monitoring of microclimatic factors, including temperature and light [35,36,37]. Therefore, elucidating the effects of seasonal environmental variation, particularly night temperature, on anthocyanin biosynthesis is critical for understanding berry coloration and quality formation in double-cropping grape production systems.

Although previous studies have revealed the effects of environmental factors on grape anthocyanin accumulation, many have focused on comparisons among different cultivars or geographical regions, making it difficult to distinguish the specific contribution of seasonal climatic variation from other environmental and management factors. Therefore, the effects of seasonal temperature variation on anthocyanin biosynthesis within the same cultivar and cultivation system remain insufficiently understood. In this study, a double-cropping system was evaluated within a single vineyard using the same cultivar ‘Summer Black’ under consistent management practices, providing a comparable framework for investigating seasonal differences in anthocyanin accumulation between summer and winter crops. This study integrates meteorological monitoring, anthocyanin profiling, and transcriptomic analysis to elucidate the association between seasonal environmental variation and anthocyanin biosynthesis in grape berries.

## 2. Materials and Methods

### 2.1. Plant Material

**Figure 1:**
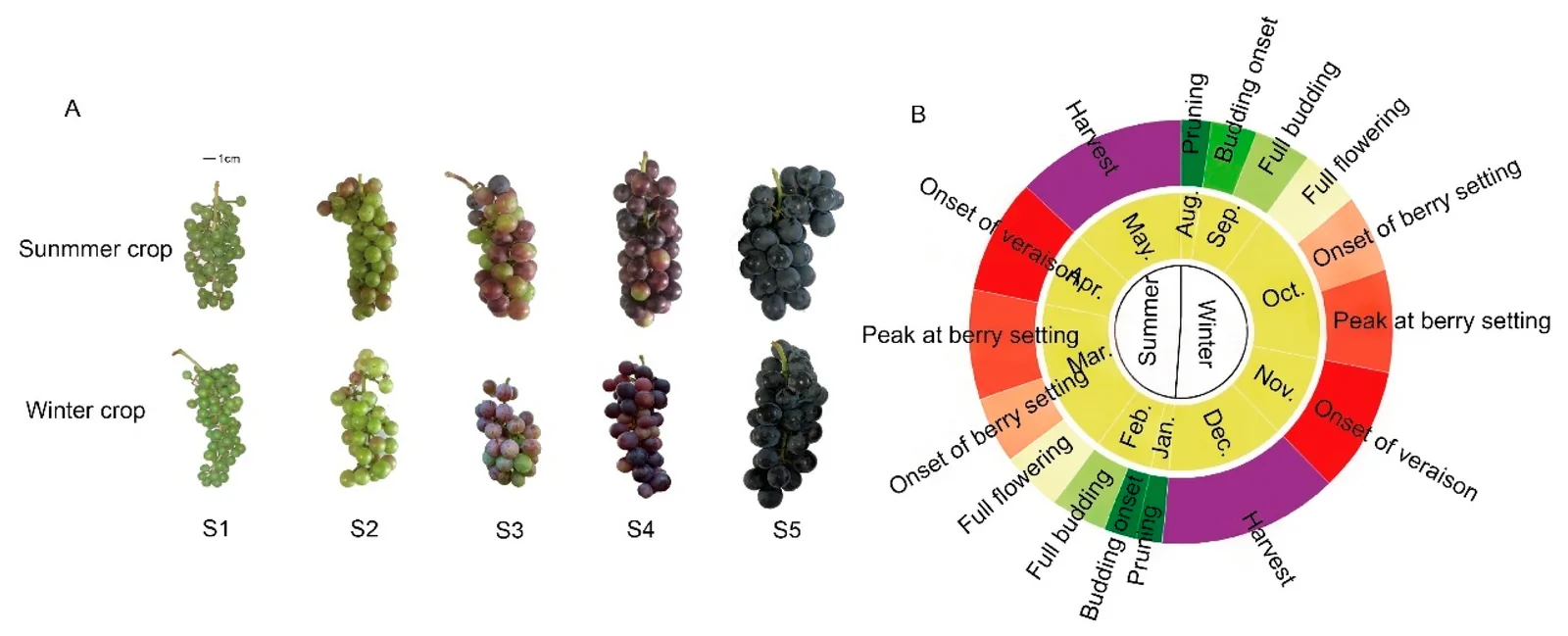
(**A**) Different growth stages of ‘Summer Black’ table grape. (**B**) Phenological diagram of the double-cropping system for ‘Summer Black’ grapes.

The research was conducted during the 2021–2022 growing season in a six-year-old commercial vineyard of ‘Summer Black’ (*Vitis vinifera* L.) located in Xiamen, Fujian Province, China (24.48° N, 118.08° E; altitude: 25 m), which is owned by Xiamen Lunong Agricultural Technology Co., Ltd. The vineyard was managed under a double-cropping system, allowing two grape harvests per year without overlapping growth cycles (Figure 1A, Table S1).

Winter grape samples were collected from 19 October to 20 December 2021. Summer grape samples were collected from 25 March to 31 May 2022. For each cropping season, five vines with similar growth vigor were selected from the same vineyard. The fruit is divided into five different developmental stages, according to the Eichhorn–Lorenz phenological scale [38]: S1, green and hard berries (E-L 31); S2, enlarged berries with reduced firmness (E-L 34); S3, enlarged berries with red coloration appearing (E-L 35); S4, fully red berries (E-L 37); and S5, mature berries with purple-black coloration (E-L 38).

At each developmental stage, six uniform clusters were collected from both sun-exposed and shaded sides of each vine. Berries were randomly collected from the shoulder, middle, and bottom regions of clusters, pooled, and separated into three independent biological replicates. The berries were immediately transported to the laboratory, where skins and pulps were carefully separated. All samples were frozen in liquid nitrogen and stored at −80 °C until further analysis. Each treatment consisted of three independent biological replicates.

### 2.2. Meteorological Data

**Figure 2:**
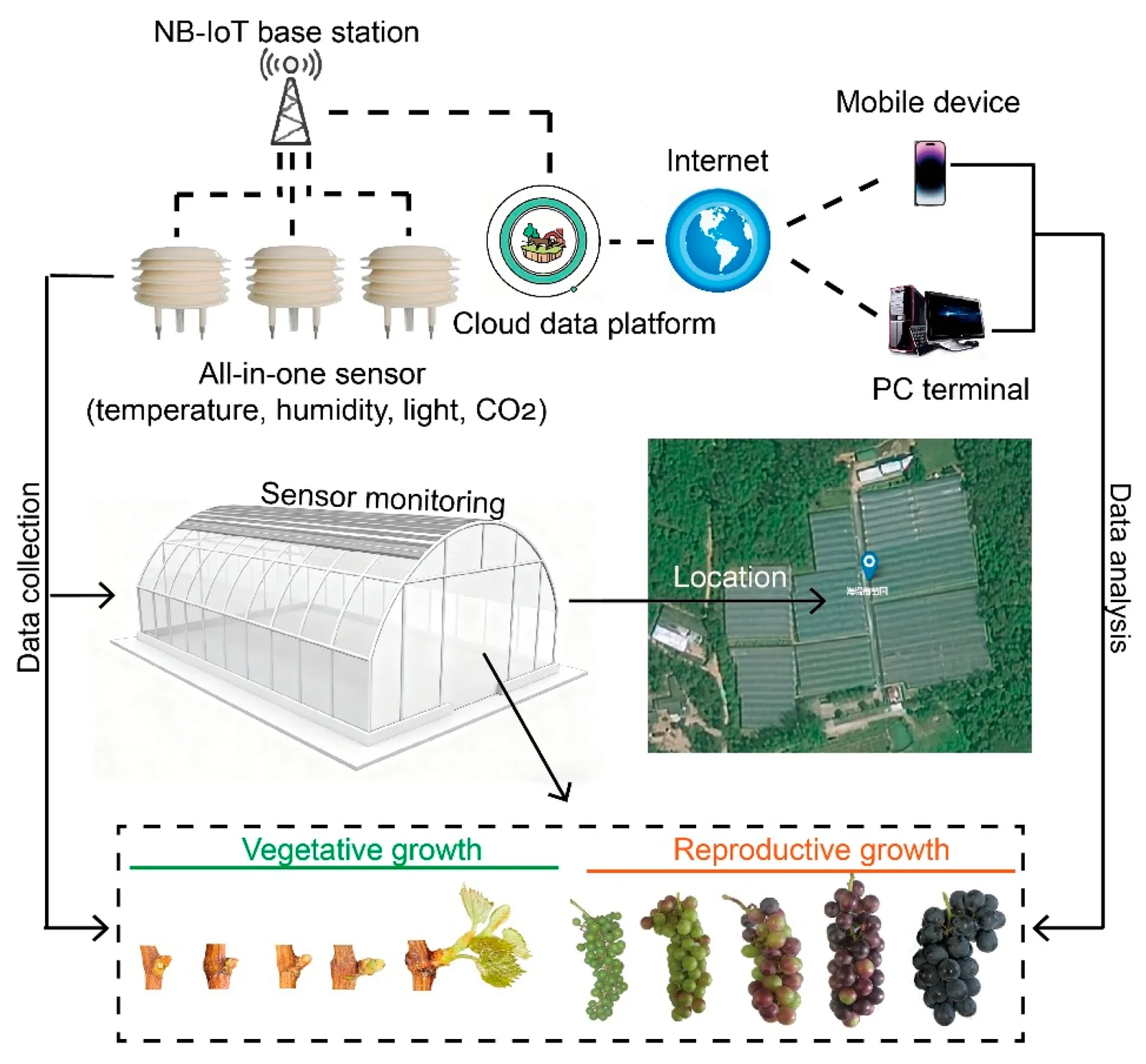
Schematic diagram of a smart sensor-based system for monitoring and regulating meteorological factors. Demonstration of the smart vineyard monitoring system, including the operational mechanism of environmental sensors, the management platform interface, the real-time IoT dashboard displaying key environmental parameters (temperature, humidity and light intensity), and the satellite view showing greenhouse distribution within the vineyard.

To accurately characterize the greenhouse microclimate, an integrated temperature–light–humidity sensor (Zhongtian IoT Co., Ltd., Weihai, Shandong, China) was installed in each of the four independent greenhouse facilities (Figure 2). The sensors were positioned between the grapevine canopy and the greenhouse framework to continuously record air temperature and light intensity during the grape growth period. Environmental data were automatically collected at 10-min intervals and transmitted to an IoT platform (http://iot.ainongye.cn/dashboard/analysis (accessed on 31 December 2022)) for data storage and subsequent analysis. Before deployment, sensors were factory-calibrated according to the manufacturer’s specifications. The measurement accuracies were ±0.1 °C for temperature, ±0.3% RH for relative humidity, and ±5% for light intensity (Table S2). The collected environmental data were checked for continuity and completeness before subsequent analyses.

Meteorological data for each phenological stage represent stage-specific averages. The analyzed parameters comprised: minimum temperature (*T*_min_, °C), maximum temperature (*T*_max_, °C), mean day temperature (*T*_day_, °C), mean night temperature (*T*_night_, °C), mean temperature (*T*_mean_, °C), diurnal temperature range (DTR, °C), mean light intensity (MLI, lx), and cumulative growing degree days (GDD, °C·d).

*T*_min_ and *T*_max_: The lowest and highest daily temperatures recorded during each phenological stage.

*T*_day_ and *T*_night_: Day temperature (07:00–17:00) and night temperature (19:00–05:00) were calculated as the mean of hourly temperature records using the following formula:

$$T\text{night}(\text{day})=\frac{1}{n}\sum_{i=1}^{n}Ti\;$$

where *Ti* is the hourly temperature measurement and n is the total number of hours (11 for day and 10 for night).

*T*_mean_: The average of all recorded temperatures during a stage, calculated as:

$$Tmean=\frac{Tmin+Tmax}{2}$$

Diurnal Temperature Range (DTR) refers to the difference between the daily maximum temperature (*T*_max_) and the daily minimum temperature (*T*_min_). It is an important climatic parameter that reflects the amplitude of daily temperature variation and is often used to assess microclimatic conditions affecting plant development.

DTR=Tmax−Tmin

MLI (Mean Light Intensity): The average photosynthetically active light intensity (lx) during the day (typically 06:00–18:00), excluding night data (lx = 0).

Growing degree day (GDD) accumulation was calculated using a base temperature (*Tb*) of 10 °C. When the daily mean temperature (*T*_mean_) was below *Tb*, it was set equal to *Tb*, resulting in no GDD accumulation for that day. The calculation followed the formula [39]:

$$\text{GDD}(\text{Accumulated})=\sum_{i=1}^{n}\left(Ti-Tb\right)$$

### 2.3. Determination of Berry Skin Color

Berry skin color was measured using a portable colorimeter (3nh Co., Ltd., Shenzhen, China). For each developmental stage, twenty berries with uniform size and color were selected. The L*, a*, and b* values were measured at three equidistant positions on the berry surface, and the average values were used for analysis.

### 2.4. Extraction and Determination of Total Anthocyanins and Anthocyanin Components

Anthocyanin profiles were analyzed following the method of Sangeeta Mitra [30] with minor modifications using an LC-100 high-performance liquid chromatography (HPLC) system (Wufeng Scientific Instruments Co., Ltd., Shanghai, China). Anthocyanin extracts were prepared using the same procedure as that described for total anthocyanin determination and filtered through a 0.45 μm microporous membrane (Biosharp, Hefei, Anhui, China) before HPLC analysis. Chromatographic separation was performed on a Brisa LC2 C18 column (4.6 mm × 250 mm, 5 μm) maintained at 45 °C. The flow rate was set at 1 mL/min, and anthocyanins were detected at 520 nm. Individual anthocyanin standard stock solutions, including delphinidin-3-O-glucoside (Dp-3-O-G), cyanidin-3-O-glucoside (Cy-3-O-G), petunidin-3-O-glucoside (Pt-3-O-G), peonidin-3-O-glucoside (Pn-3-O-G), and malvidin-3-O-glucoside (Mv-3-O-G), were purchased from Nanjiang Biotechnology Co., Ltd. (Fuzhou, China). Standard solutions were prepared by dissolving the compounds in 2% HCl-methanol solution and adjusting the volume to 1 mL. The stock solution was diluted to prepare a series of five standard mixed samples with concentrations ranging from 200 to 1000 ppm. The column temperature was set to 45 °C, and the flow rate was 1 mL/min. The mobile phase A comprised water, formic acid, and acetonitrile in a ratio of 40:10:50, whereas mobile phase B consisted of water, acetonitrile, and formic acid in a ratio of 87:10:3. The gradient schedule comprised the following phases: 10% A (0–0 min), 25% A (0–10 min), 31% A (10–15 min), 40% A (15–20 min), and 10% A (20–25 min). Individual anthocyanin components were identified according to their retention times compared with authentic standards. Quantification was performed based on the corresponding calibration equations, and the results were expressed as μg/g fresh weight (FW).

### 2.5. RNA-Seq Library Preparation and Sequencing

‘Summer Black’ grape skins were collected in winter during three developmental stages (S1, S3, and S5). These transcriptome samples were collected from winter berries to characterize developmental changes associated with anthocyanin accumulation. The skins were standardized at each phase and divided into three biological replicates. Each sample weighed 20 g, was instantly frozen in liquid nitrogen, and then stored at −80 °C until RNA sequencing. The samples were shipped on dry ice to Biomarker Technologies Corporation in Qingdao, China, for transcriptome sequencing.

Clean data from quality filtering were aligned to the *Vitis vinifera* reference genome (ftp://ftp.ensemblgenomes.org/pub/plants/release-45/fasta/vitis_vinifera/ (accessed on 13 July 2026)) using the HISAT2 alignment algorithm. Following alignment, StringTie was utilised to assemble and quantify the mapped reads. Gene expression levels from several sample groups were used for differential expression analysis, functional annotation of differentially expressed genes (DEGs), and enrichment analysis.

### 2.6. qRT-PCR Validation of Transcriptome-Identified Candidate Genes

Selected candidate genes identified from transcriptomic analysis were further validated by qRT-PCR using berry skins collected from both summer and winter crops at three representative developmental stages (S1, S3, and S5). Total RNA was extracted using a commercial kit from TIANGEN (Beijing, China), with three biological replicates per sample. qRT-PCR reactions were performed using Hieff qPCR SYBR Green Master Mix (Yeasen, Shanghai, China) according to the manufacturer’s instructions. Each 20 μL reaction contained 0.4 μM gene-specific primers. RNA integrity was verified by RNase-free agarose gel electrophoresis, and RNA concentration and purity were assessed using a Quawell Q5000 spectrophotometer (Quawell, Sunnyvale, CA, USA). Gene-specific primers were designed using Primer 5 software. The qRT-PCR thermal cycling conditions were as follows: initial denaturation at 95 °C for 5 min followed by 40 cycles of 95 °C for 10 s and 60 °C for 30 s. *Actin* was used as the internal reference gene. Gene expression levels were calculated using the 2^–ΔΔCt^ method.

### 2.7. Statistical Analysis

All data are presented as the mean ± standard deviation (SD) of three biological replicates. Statistical analyses were performed using SPSS 26.0 software. For datasets involving two experimental factors (cropping season and developmental stage), two-way analysis of variance (ANOVA) was performed to evaluate the effects of cropping season, developmental stage, and their interaction, followed by Tukey’s multiple comparison test. Differences were considered significant at *p* < 0.05 and highly significant at *p* < 0.01. Pearson’s correlation coefficients were calculated to assess the relationships between variables using SPSS 26.0. The correlations were visualized as three-dimensional (3D) scatter plots using Origin 2023 software. The R package “Mfuzz” was employed to conduct gene expression trend analysis of differentially expressed genes (DEGs) across various developmental stages.

## 3. Results

### 3.1. Temperature and Light Data

**Figure 3:**
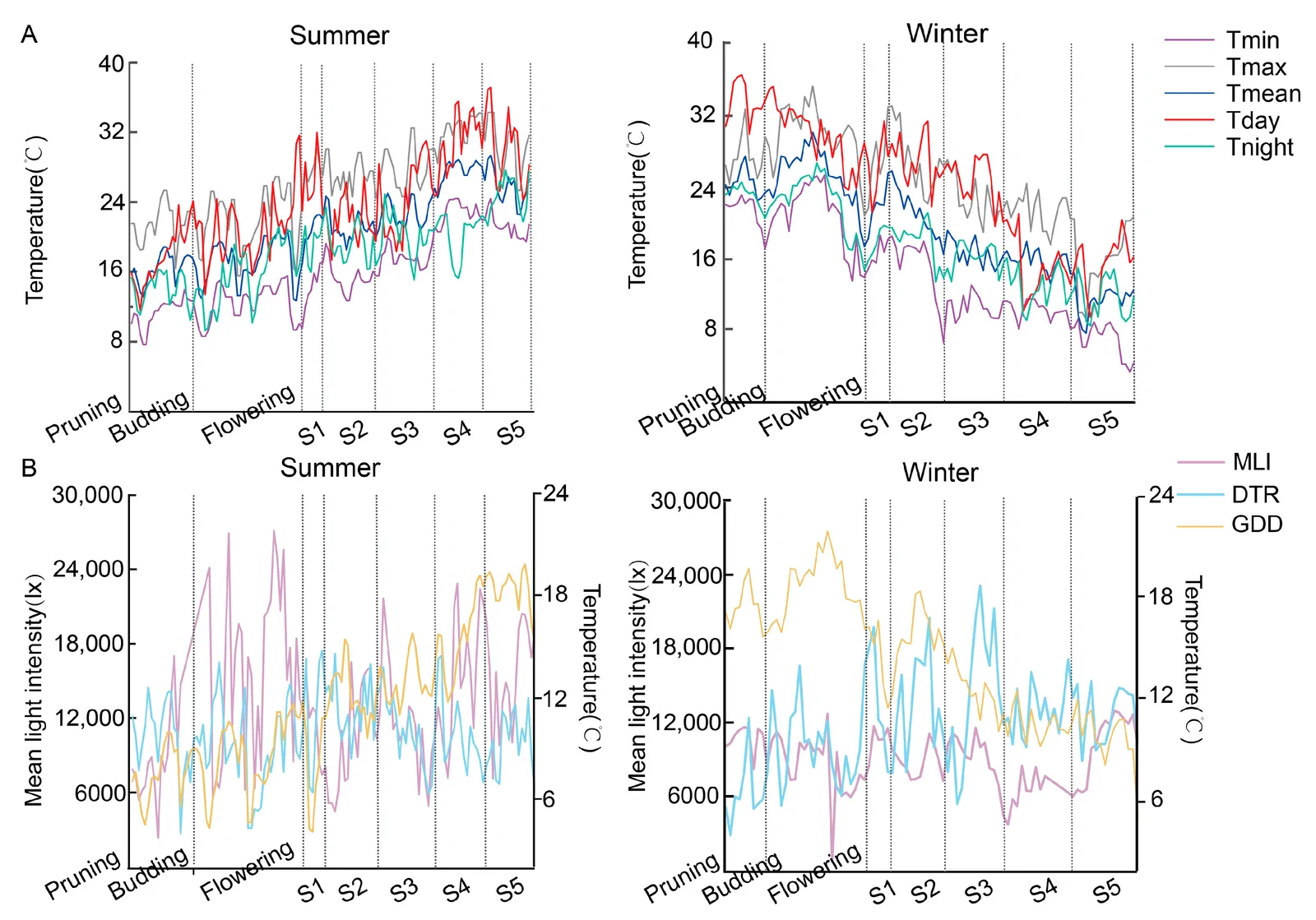
Meteorological factors at different developmental stages of winter and summer crops under the double-cropping system. (**A**) Temperature trends across developmental stages. (**B**) Changes in mean light intensity (MLI), diurnal temperature range (DTR), and growing degree days (GDD) during berry development.

Temperature and light conditions showed clear seasonal differences during ‘Summer Black’ grape berry development (Figure 3). The mean daily minimum (*T*_min_) and maximum (*T*_max_) temperatures increased during summer berry development but decreased during winter development (Figure 3A). The daily mean temperature (*T*_mean_) reached 27.9 °C in summer berries but declined to 14.8 °C in winter berries. In contrast, the diurnal temperature range (DTR) increased during winter berry development but showed a decreasing trend in summer berries (Figure 3B). Light conditions also differed between seasons, with higher mean light intensity (MLI) and growing degree days (GDD) recorded in summer berries. In addition, both day and night temperatures were consistently higher during summer cultivation, whereas they gradually declined throughout winter berry development.

### 3.2. Comparison of Fruit Skin Color and Anthocyanin Composition at Different Developmental Stages

**Figure 4:**
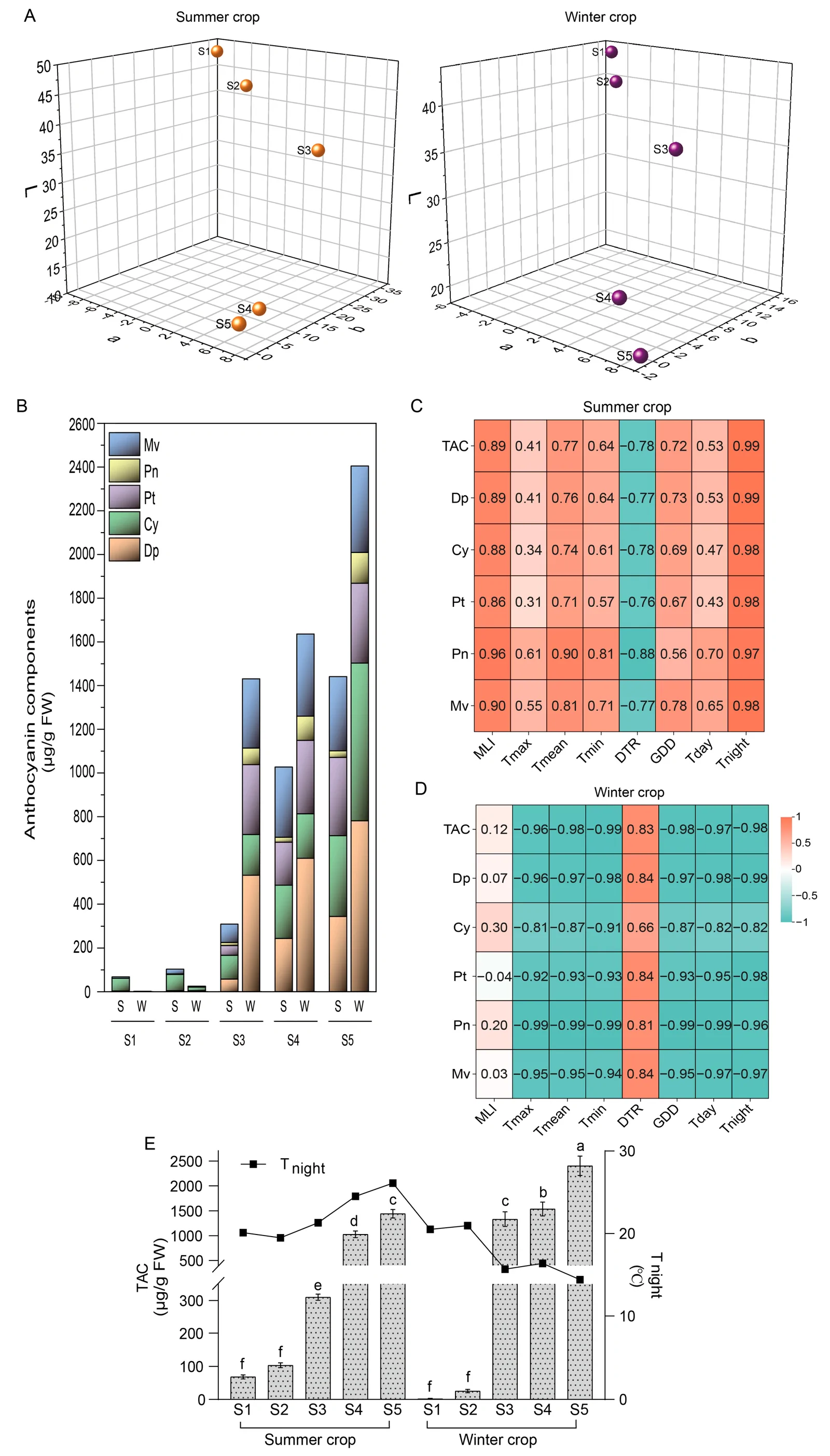
(**A**) The color changes in winter and summer grape skin in the five stages visualized as a three-dimensional (3D) scatter plot. There were three L, a, and b dimensions: brightness (L*), red-green (a*), and yellow-blue (b*). Points denote stages. (**B**) The composition of anthocyanins differed between summer and winter fruits across developmental stages (S, summer; W, winter). (**C**,**D**) Correlations between anthocyanin composition and meteorological factors in summer- (**C**) and winter-cultivated (**D**) grapevines. (**E**) Changes in total anthocyanin content (TAC) and nighttime temperature during berry development in summer and winter crops. Bars represent TAC (μg/g FW), and the line represents nighttime temperature. Data represent mean ± SD of *n* = 3 biological replicates. Two-way ANOVA was performed to evaluate the effects of cropping season, developmental stage, and their interaction on TAC accumulation, followed by Tukey’s multiple comparison test. Different letters indicate significant differences among season × developmental stage combinations (*p* < 0.05). Pearson correlation coefficients were calculated based on five developmental stages (*n* = 5). Minimum daily temperature (*T*_min_); maximum daily temperature (*T*_max_); mean daily temperature (*T*_mean_); diurnal temperature range (DTR); mean Light Intensity (MLI); growing degree days (GDD); day temperature (*T*_day_); night temperature (*T*_night_); delphinidin (Dp); cyanidin (Cy); petunidin (Pt); peonidin (Pn); malvidin (Mv); total anthocyanins (TAC).

Changes in berry skin color during fruit development are shown in Figure 4A. The L* values of both summer and winter berries decreased progressively during ripening, indicating a gradual darkening of the skin. Compared with summer berries, winter berries exhibited lower L* values and higher a* values at the late developmental stages, suggesting deeper red coloration. Meanwhile, b* values declined during ripening and became negative in winter berries at stage S5, indicating the appearance of bluish pigmentation.

Anthocyanin accumulation in grape skins showed significant variation between summer and winter grapes (Figure 4B). The calibration curves showed good linearity, with correlation coefficients (R^2^) ranging from 0.9907 to 0.9995 (Table S3). During fruit ripening, the anthocyanin components delphinidin (Dp), cyanidin (Cy), petunidin (Pt), peonidin (Pn), and malvidin (Mv) exhibited significant increases. Winter berries exhibited a faster accumulation rate and consistently higher anthocyanin levels from veraison (S3) onward compared with summer berries. At maturity (S5), the total anthocyanin content in winter berries reached 2405.38 ± 195.05 μg/g FW, which was approximately 1.7-fold higher than that in summer berries (1440.94 ± 85.81 μg/g FW). Among the individual anthocyanins, Dp and Cy were the predominant components and showed markedly higher concentrations in winter berries. At the veraison stage (S3), the Mv content in winter berries (316.98 ± 0.60 μg/g FW) was approximately fourfold higher than that in summer berries (83.70 ± 0.91 μg/g FW). In addition, Pt accumulation in winter berries began earlier and increased sharply from veraison onward. Although Pn accounted for the smallest proportion of total anthocyanins, its content in winter berries at S5 (140.71± 1.70 μg/g FW) remained significantly higher than in summer berries (*p* < 0.05). Notably, total anthocyanin concentration in winter berries at S3 was approximately 57-fold higher than at S2 (*p* < 0.05), indicating that veraison represents a critical stage for anthocyanin accumulation.

### 3.3. Correlation Analysis Between Anthocyanin Accumulation and Meteorological Factors Across Different Growth Cycles

The correlations between meteorological factors and anthocyanin accumulation are shown in Figure 4C,D. During summer cultivation, TAC and individual anthocyanin components exhibited strong positive correlations with nighttime temperature (*T*_night_) (r = 0.99, *p* < 0.05), followed by mean light integral (MLI) (r = 0.89, *p* < 0.05). Among the anthocyanin components, cyanidin (Cy) showed the strongest correlation with *T*_night_ (r = 0.98, *p* < 0.05).

In contrast, winter berries exhibited strong negative correlations between TAC and temperature-related variables, including *T*_max_, *T*_mean_, *T*_min_, *T*_day_, *T*_night_, and GDD (r < −0.97, *p* < 0.05). Among these factors, *T*_min_ showed the strongest correlation with TAC (r = −0.99, *p* < 0.05), while TAC was positively correlated with the diurnal temperature range (DTR) (r = 0.83, *p* < 0.05). Among the individual anthocyanins, delphinidin (Dp) exhibited the strongest correlation with *T*_night_ (r = −0.99, *p* < 0.05), followed by petunidin (Pt) and malvidin (Mv). These results indicate that night temperature is a key environmental factor influencing anthocyanin accumulation in grape berries. To further illustrate the relationship between night temperature and anthocyanin accumulation during berry development, the dynamic changes in TAC and night temperature across developmental stages are presented in Figure 4E. During summer cultivation, night temperature gradually increased from veraison to maturity, accompanied by a progressive increase in TAC. In contrast, winter berries developed under substantially lower night temperatures, particularly during the ripening stages (S3–S5), which coincided with markedly higher TAC levels compared with summer berries. Notably, winter ripening occurred under night temperatures of approximately 14.0–16.0 °C, whereas summer berries experienced warmer nights of approximately 24.0–26.0 °C.

### 3.4. Expression Patterns of Anthocyanin Biosynthesis Genes and Their Associations with Meteorological Factors

**Figure 5:**
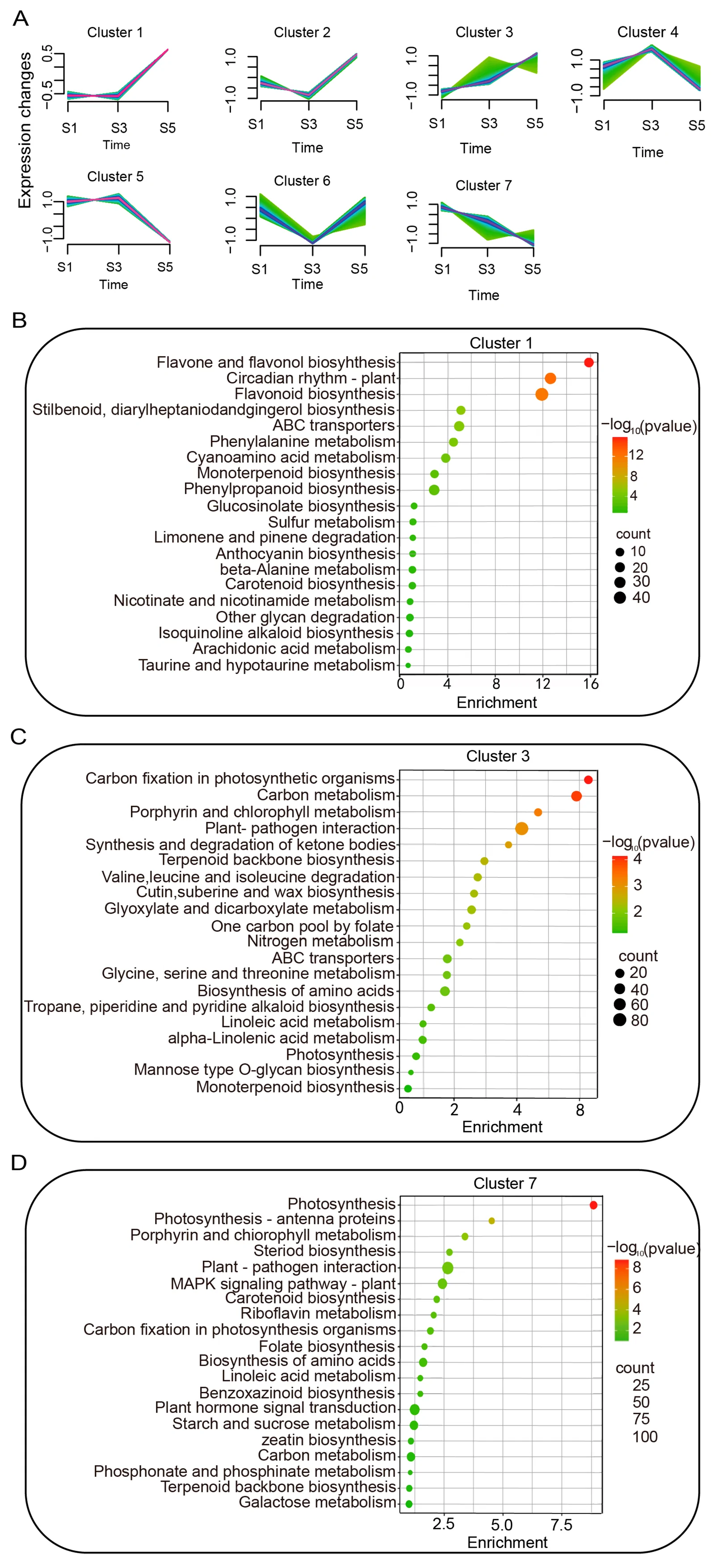
(**A**) Gene expression trends at S1, S3 and S5 stages. The horizontal axis represents developmental stages, and the vertical axis indicates the magnitude of gene expression changes. Positive and negative values indicate gene up-regulation and down-regulation, respectively. (**B**–**D**) Bubble plots showing KEGG pathway enrichment analysis for different gene clusters.

Transcriptome sequencing was conducted on the berry skins of winter-cultivated ‘Summer Black’ grapes at three developmental stages (S1, S3, and S5), with three biological replicates for each sample. A trend analysis of differentially expressed genes (DEGs) in grape berry skin at various developmental stages generated seven distinct expression clusters (Figure 5A). Clusters 1 and 3 showed expression patterns consistent with anthocyanin accumulation from the green stage to ripening, suggesting that genes in these clusters may be involved in anthocyanin biosynthesis. In contrast, Cluster 7 displayed an opposite expression pattern to anthocyanin accumulation, indicating that genes in this cluster may be negatively associated with anthocyanin accumulation or involved in other developmental processes. The expression patterns of Clusters 2, 4, 5, and 6 did not correspond to the changes in anthocyanin content, suggesting their potential involvement in other biological processes during berry skin development.

Cluster 1 comprises 871 DEGs with low expression at the S1 and S3 stages and elevated expression at ripening. KEGG analysis revealed (Figure 5B) enrichment in ‘Flavonoid biosynthesis’, ‘Flavone and flavonol biosynthesis’, and ‘Phenylalanine metabolism’, including key structural genes (*PAL*, *CHS*, *CHI*, *F3′5′H*) and regulatory transcriptome factors (MYB, bHLH, WRKY, bZIP, C2H2, LOB).

Cluster 3 includes 1196 DEGs with a continuous increase in expression during berry skin development (Figure 5C). These genes are enriched in ‘Carbon fixation’, ‘Carbon metabolism’, and ‘ABC transporters’, and include anthocyanin-related structural genes (*C4H*, *4CL*, *ANS*, *DFR*, *UFGT*) and transcriptome factors from the bHLH, WRKY, MYB, bZIP, and NAC families.

Cluster 7 consists of 1945 DEGs with a declining expression trend during berry development, opposite to Cluster 3. These genes are enriched in ‘Carotenoid biosynthesis’, ‘MAPK signaling pathway—plant’, ‘Carbon metabolism’, and ‘Starch and sucrose metabolism’ (Figure 5D). The cluster includes regulatory genes from the C2H2, CO-like, AP2/ERF, B3, HB-HD-ZIP, and MADS-MIKC families, along with anthocyanin-related structural genes (*PAL*, *4CL*, *CHI*, *DFR*).

**Figure 6:**
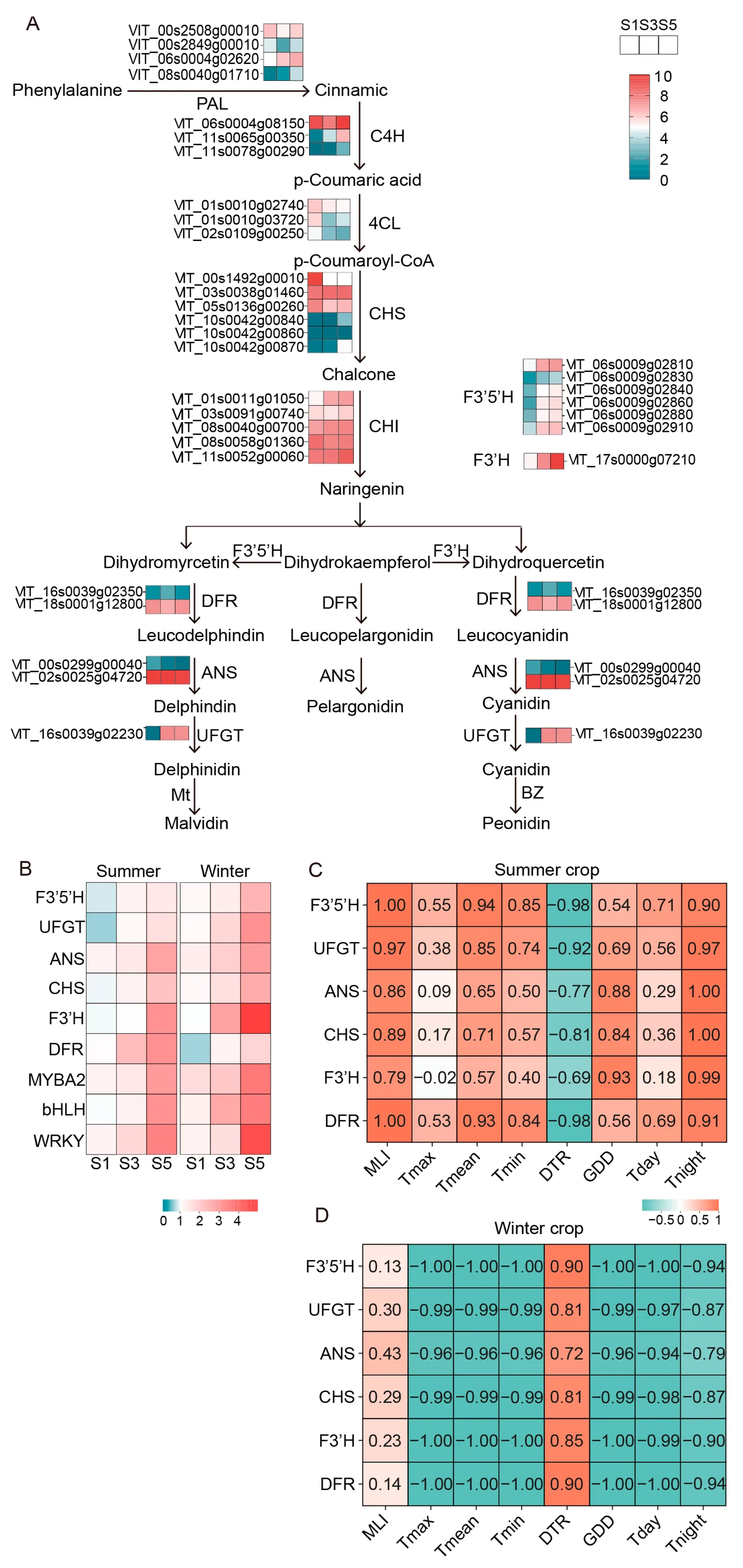
(**A**) Heatmap showing the expression of candidate flavonoid pathway genes involved in anthocyanin synthesis across three fruit developmental stages (S1, S3, and S5) as determined by RNA-seq. Colors in the heatmap represent log_2_(FPKM + 1) values, with darker red indicating higher gene expression. (**B**) Expression heatmap of candidate genes based on qRT-PCR analysis. qRT-PCR analyses were performed on three biological replicates. Color intensity represents relative gene expression levels, with darker shades indicating higher expression. (**C**,**D**) Associations between the expression levels of key anthocyanin biosynthesis genes and meteorological factors during summer and winter cultivation. Numbers in the heatmap represent Pearson’s correlation coefficients (*r*) between variables. Pearson correlation coefficients were calculated based on three developmental stages (S1, S3, and S5; *n* = 3). Positive and negative values indicate positive and negative associations, respectively, and the absolute value of r represents the strength of the linear relationship. Colors represent the strength and direction of correlations, with red shades indicating positive correlations, blue-green shades indicating negative correlations, and lighter colors representing weaker correlations. Minimum daily temperature (*T*_min_); maximum daily temperature (*T*_max_); mean daily temperature (*T*_mean_); diurnal temperature range (DTR); Mean Light Intensity (MLI); growing degree days (GDD); day temperature (*T*_day_); night temperature (*T*_night_).

Based on the expression patterns of DEGs and KEGG enrichment analysis, key enzyme genes involved in anthocyanin biosynthesis in grapes were identified and subjected to expression profile analysis. Expression analysis of key anthocyanin biosynthesis genes showed that *PAL*, *C4H*, *CHS*, *CHI*, *UFGT*, and *F3′H* gradually increased during fruit development, whereas *4CL* and *DFR* decreased, and *ANS* and *F3′5′H* exhibited an increasing expression trend (Figure 6A).

The relative expression levels of six structural genes (*F3′5′H*, *F3′H*, *UFGT*, *ANS*, *CHS*, and *DFR*) and three regulatory genes (*MYBA2*, *WRKY75*, and *bHLH*) were analyzed by qRT-PCR in grape berries from two cropping seasons at three developmental stages (Figure 6B). The qRT-PCR results showed expression patterns largely consistent with those obtained from the RNA-seq analysis. Across the three developmental stages, the expression levels of all nine genes increased during fruit development and reached their highest levels at the ripening stage. Furthermore, *F3′H*, *UFGT*, *ANS*, *CHS*, *MYBA2*, *WRKY75*, and *bHLH* exhibited significantly higher expression levels in winter berries than in summer berries, whereas *F3′5′H* and *DFR* showed significantly lower expression levels in winter berries.

Correlation analysis (Figure 6C,D) revealed that night temperature (*T*_night_) showed strong associations with the expression patterns of several anthocyanin biosynthesis genes, including *CHS*, *F3′H*, *DFR*, and *UFGT*. These correlations suggest that nocturnal thermal conditions may represent an important environmental factor associated with the transcriptional variation in anthocyanin-related genes. During summer cultivation, mean light integral (MLI) showed positive associations with several anthocyanin biosynthesis genes, including *F3′5′H* (r = 1.0), *DFR* (r = 1.0), and *UFGT* (r = 0.97), suggesting that light availability may be associated with transcriptional changes in these structural genes. In contrast, the diurnal temperature range (DTR) showed negative associations with the expression of several anthocyanin-related genes. Under winter cultivation conditions, temperature-related factors exhibited stronger associations with gene expression patterns. Night temperature (*T*_night_) and minimum temperature (*T*_min_) showed negative associations with *CHS* (r = −1.00, *n* = 3), *DFR* (r = −1.00, *n* = 3), *ANS* (r = −1.00, *n* = 3), and *UFGT* (r = −0.90, *n* = 3), suggesting a potential relationship between cooler thermal conditions and the expression patterns of these genes. Meanwhile, DTR showed positive associations with *F3′H* (r = 0.90, *n* = 3) and *DFR* (r = 0.90, *n* = 3). In comparison, MLI showed relatively weaker associations with gene expression during winter.

## 4. Discussion

### 4.1. Seasonal Temperature Variation and the Contribution of Night Temperature to Anthocyanin Accumulation

Seasonal fluctuations in growth conditions between summer and winter result in varied patterns and levels of anthocyanin accumulation and composition [40]. The rapid accumulation of anthocyanins in grape berries primarily occurs from veraison to ripening, a period defined by viticulturists as 1 to 3 weeks after the onset of ripening when berry color shifts from green to yellow or red [22]. Consistent with previous reports [25], anthocyanin accumulation increased rapidly during the transition from veraison to ripening in the present study, confirming that this developmental stage represents a critical period for pigment biosynthesis and is highly responsive to environmental cues. Temperature is widely recognized as a key environmental factor affecting anthocyanin biosynthesis in grapes. Numerous studies have reported that relatively low temperatures favor anthocyanin accumulation, whereas high temperatures suppress pigment formation [26,41,42]. Under double-cropping systems, cooler winter conditions have been shown to enhance anthocyanin concentrations compared with summer production [33]. Correlation analysis further revealed that both total anthocyanin content and individual anthocyanin components were strongly associated with night temperature. Notably, berries ripening under cooler nocturnal conditions during winter showed markedly higher anthocyanin accumulation than those developing under warmer summer nights, suggesting that night temperature may contribute to seasonal variation in pigment accumulation. However, seasonal differences in berry coloration are likely influenced by multiple environmental and physiological factors. Although nighttime temperature showed strong associations with anthocyanin accumulation and related gene expression, these differences may also reflect the combined effects of light availability, temperature regimes, and crop developmental conditions under double-cropping systems.

### 4.2. Temperature-Associated Variation in Anthocyanin Composition Is Linked to F3′H/F3′5′H Expression

Seasonal differences in temperature not only affected total anthocyanin concentration but also altered the composition of anthocyanin derivatives. The balance between dihydroxylated and trihydroxylated anthocyanins is largely determined by the relative activities of flavonoid 3′-hydroxylase (*F3′H*) and flavonoid 3′,5′-hydroxylase (*F3′5′H*), which control key branch points in the flavonoid biosynthetic pathway.

Under relatively warm summer conditions, higher night temperatures during berry ripening were associated with increased accumulation of dihydroxylated anthocyanins such as cyanidin derivatives. Previous studies have reported that elevated temperatures can inhibit the activity or expression of *F3′5′H*, thereby restricting the biosynthesis of trihydroxylated anthocyanins including delphinidin- and malvidin-derived pigments [43,44]. Consistent with these observations, the relatively lower expression of *F3′5′H* compared with *F3′H* under summer conditions may explain the preferential accumulation of cyanidin-derived pigments.

In contrast, cooler environmental conditions during winter cultivation were associated with a pronounced shift in anthocyanin composition toward trihydroxylated forms, including delphinidin-, malvidin-, and petunidin-derived pigments, which contribute to deeper bluish-purple berry coloration.

Consistent with our transcriptomic analysis, several structural genes involved in anthocyanin biosynthesis exhibited temperature-dependent expression patterns (Table S4). Previous studies have reported that genes such as *CHS*, *F3H*, *F3′5′H*, and *DFR* are often induced under relatively low-temperature conditions, whereas *F3′H* tends to show enhanced expression under elevated temperatures. Such differential temperature responsiveness of key pathway enzymes may contribute to the seasonal shifts in anthocyanin composition observed in the present study.

### 4.3. Transcriptomic Insights into Temperature-Associated Regulation of Anthocyanin Biosynthesis

Transcriptome enrichment analysis revealed that flavonoid biosynthesis and phenylpropanoid metabolism were among the major pathways associated with berry development under winter conditions. These results provide a molecular framework for interpreting the seasonal differences observed in anthocyanin accumulation. Several structural genes involved in anthocyanin biosynthesis, including *CHS*, *DFR*, *ANS*, and *UFGT*, showed dynamic expression patterns during ripening, indicating coordinated regulation of the pathway rather than regulation by a single branch enzyme. Notably, *F3′5′H* exhibited higher expression during winter ripening, which was consistent with the increased accumulation of trihydroxylated anthocyanins. This result suggests that enhanced *F3′5′H* expression under cooler winter conditions may contribute to the increased accumulation of trihydroxylated anthocyanins. Similar responses have been reported in grape berries subjected to night-cooling treatments during veraison, where increased expression of *CHS* and *UFGT* was accompanied by enhanced anthocyanin accumulation [22]. Temperature-mediated regulation of anthocyanin biosynthesis likely involves complex transcriptional and hormonal regulatory networks. Previous studies have shown that temperature signals can influence the activity of MYB transcription factors that regulate structural genes in the flavonoid pathway [45]. During summer berry development, higher temperature and strong light intensity were associated with altered expression patterns of regulatory genes such as *MYBA2*, *WRKY75*, and *bHLH*, which may contribute to reduced expression of downstream structural genes such as UFGT and decreased anthocyanin accumulation in grape berries. In particular, abscisic acid (ABA) has been reported to be involved in grape berry ripening and anthocyanin accumulation. Exogenous ABA application and night temperature were shown to influence anthocyanin composition in ‘Pinot noir’ grapes, suggesting that ABA signaling may interact with temperature responses during berry maturation [27]. However, the precise molecular mechanisms underlying ABA-mediated regulation of anthocyanin biosynthesis under different temperature conditions remain to be further elucidated.

In contrast, cooler winter conditions, particularly lower night temperatures during ripening (14.4–15.7 °C), were associated with enhanced accumulation of trihydroxylated anthocyanins, including delphinidin-, malvidin-, and petunidin-derived pigments, contributing to the darker bluish-purple coloration of berries. Transcriptome enrichment analysis revealed significant enrichment of the flavonoid biosynthesis pathway during winter berry development. Several structural genes involved in anthocyanin biosynthesis, including *CHS*, *DFR*, *ANS*, and *UFGT*, showed dynamic expression patterns in RNA-seq analysis. These candidate genes were further validated by qRT-PCR in both summer and winter berries, supporting their potential involvement in seasonal variation in anthocyanin accumulation. This observation is consistent with previous reports showing that night-cooling treatments during veraison enhanced *CHS* and *UFGT* expression and promoted anthocyanin accumulation [22].

Overall, the present study demonstrates that seasonal temperature differences under a double-cropping system play an important role in shaping anthocyanin accumulation and composition in grape berries. Compared with summer cultivation, the cooler thermal conditions during winter, particularly lower night temperatures, were more favorable for anthocyanin biosynthesis and resulted in higher pigment accumulation and darker berry coloration. Transcriptomic analysis further indicated that these temperature differences were associated with changes in the expression of key structural genes involved in the anthocyanin pathway. Although seasonal variation involves multiple environmental factors, the consistent association between night temperature and anthocyanin accumulation observed in this study suggests that nocturnal thermal conditions represent an important component of seasonal regulation of berry coloration. These findings highlight the importance of thermal conditions during berry ripening and suggest that managing temperature environments, particularly night temperature, may represent an effective strategy for improving grape berry coloration. Continuous microclimate monitoring using IoT-based sensors may therefore provide a useful tool for optimizing temperature conditions in double-cropping grape production systems.

## 5. Conclusions

In double-cropped ‘Summer Black’ grapes, winter berries accumulated nearly twice the total anthocyanin content observed in summer berries. Seasonal differences in thermal conditions, particularly night temperature during the ripening period, were associated with distinct patterns of anthocyanin accumulation and composition. Lower night temperatures in winter were associated with higher *F3′5′H* expression and increased accumulation of trihydroxylated anthocyanins, particularly delphinidin-derived pigments, contributing to deeper purplish-black berry coloration. In contrast, warmer summer conditions were associated with relatively higher *F3′H* expression and greater accumulation of dihydroxylated anthocyanins, particularly cyanidin derivatives. Transcriptomic analysis further indicated that seasonal thermal variation was accompanied by coordinated changes in genes involved in the flavonoid biosynthetic pathway, suggesting that thermal conditions may influence anthocyanin formation through modulation of key structural genes involved in anthocyanin biosynthesis. These findings highlight the importance of managing thermal environments, particularly night temperature, during berry ripening to improve anthocyanin accumulation and fruit coloration in double-cropping grape production systems.

## Data Availability

The raw RNA-seq data generated in this study have been deposited in the NCBI Sequence Read Archive (SRA) database under BioProject accession number PRJNA1493293. The data presented in this study are available in the article and Supplementary Materials. Further inquiries can be directed to the corresponding author.

## Supplementary Materials

The following supporting information can be downloaded at: https://www.mdpi.com/article/10.3390/plants15162501/s1, Table S1. Sensor performance and parameters. Table S2. Time periods of phenological stages for temperature and light data collection. Table S3. Calibration curves and retention times of anthocyanin standards. Table S4. Temperature-dependent gene involved in anthocyanin biosynthesis.

## Abbreviations

The following abbreviations are used in this manuscript:

ABA	Abscisic acid
ANOVA	Analysis of variance
ANS	Anthocyanidin synthase
CHS	Chalcone synthase
Cy	Cyanidin
DEG(s)	Differentially expressed gene(s)
DFR	Dihydroflavonol 4-reductase
Dp	Delphinidin
DTR	Diurnal temperature range
F3′H	Flavonoid 3′-hydroxylase
F3′5′H	Flavonoid 3′,5′-hydroxylase
FPKM	Fragments per kilobase of transcript per million mapped reads
FW	Fresh weight
GDD	Growing degree days
L*	Lightness
MLI	Mean light intensity
Mv	Malvidin
Pn	Peonidin
Pt	Petunidin
qRT-PCR	Quantitative reverse transcription PCR
RNA-seq	RNA sequencing
SD	Standard deviation
TAC	Total anthocyanin content
*T*_day_	Day temperature
*T*_mean_	Mean daily temperature
*T*_night_	Night temperature
*T*_max_	Maximum daily temperature
*T*_min_	Minimum daily temperature
UFGT	UDP-glucose:flavonoid 3-O-glucosyltransferase
